# Evaluation of the analgesic potential and safety of *Cinnamomum camphora* chvar. *Borneol* essential oil

**DOI:** 10.1080/21655979.2021.1996149

**Published:** 2021-12-09

**Authors:** Shanshan Xiao, Hang Yu, Yunfei Xie, Yahui Guo, Jiajia Fan, Weirong Yao

**Affiliations:** a State Key Laboratory of Food Science and Technology, Jiangnan University, Wuxi, Jiangsu Province, China; b School of Food Science and Technology, Jiangnan University, Wuxi, Jiangsu Province, China; c Collaborative Innovation Center of Food Safety and Quality Control in Jiangsu Province, Jiangnan University, Wuxi, Jiangsu Province, China; d Research and Development Department, Chunjingziran Biotechnology Co. Ltd, Shaoxing, Zhejiang Province, China

**Keywords:** Acute toxicity, analgesia, eye irritation, pain-related factor, skin irritation

## Abstract

*Cinnamomum camphora* chvar. *Borneol* essential oil (BEO, 18.2% v/v borneol) is a by-product of steam distillation to produce natural crystalline borneol (NCB, 98.4% v/v borneol). Given the known medicinal properties of borneol, the analgesic function and safety were studied. Horn’s method and the Draize test revealed a gender difference in mice regarding acute oral LD_50_, i.e., low-toxicity to female mice (2749 mg/kg), but practically nontoxic to male mice (5081 mg/kg). There was no acute and skin or eye irritation when BEO was applied directly, if the BEO concentration was less than 50%. The analgesic effect of BEO was evaluated by the glacial acetic acid-induced writhing pain model. Continuous topical application of BEO to the abdomen of mice for 6 d, significantly reduced observed writhing in mice (*p* < 0.001) with a strong dose-response relationship (r = −0.9006). Concomitantly, the levels of the serum pain-related mediators, prostaglandin E_2_ (PGE_2_) and transient receptor potential melastatin-8 (TRPM8) were significantly reduced (*p* < 0.001), and the latter showed a strong dose-response relationship (r = −0.9427). Therefore, BEO had similar analgesic functions to borneol and was demonstrated to be safe for medicinal use.

## GRAPHICAL ABSTARCT

**Figure:**
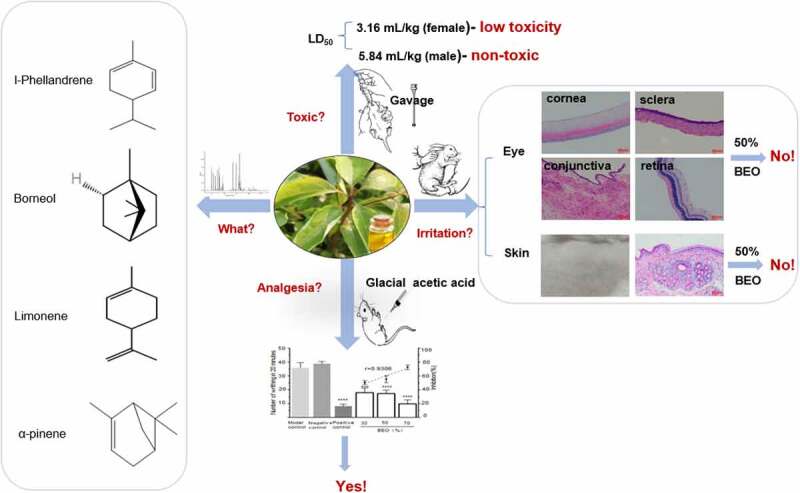

## 1. Introduction

Borneol is a valuable, high-grade flavor and pharmaceutical raw material, which is approved by the US Food and Drug Administration as a ‘generally recognized as safe’ food flavor compound [1]. It can also be used as a fragrance ingredient in cosmetics, perfumes, shampoos, soaps and household cleaners [1]. Borneol comes from two sources: chemical synthesis and extraction of plant material by steam distillation, mainly *Cinnamomum camphora* chvar. *Borneol* (*C. camphora*) branches and leaves. Natural borneol is d-borneol and is widely used in China and Southeast Asia, not only in food but also in folk medicine. Moreover, it is an important component of 63 Chinese herbal medicines that can reduce pain and swelling [1].

In recent ten years, *C. camphora* has been planted on a large scale in several provinces of Jiangxi, Zhejiang, Jiangsu and other regions in China, the leaves and branches are rich in borneol. When fresh leaves and branches of *C. camphora* are industrially steam-distilled, two products were obtained at the same time, including natural borneol and BEO, water vapor with volatile substances crystallizes (natural borneol) on the inner wall when passing through the condenser, The remaining volatile components condensed into water with steam, there was a layer of essential oil floating on the water, we called it as borneol essential oil (BEO), its main compound was borneol with about 18.2% (v/v), and there are also many other volatile components present, including α-pinene, l-Phellandrene, limonene, β-pinene, camphene, caryophyllene oxide, sabinene and linalool [2]. Most of the commercially available borneol is produced by chemical synthesis, with no BEO as a by-product. Consequently, it is important to explore the biological properties of BEO.

There have been many reports relating to the biological properties of borneol, as well as to other components found in BEO and other essential oils containing similar components to those in BEO. Borneol significantly reduced hyperalgesia in mice with chronic inflammatory pain and neuropathic pain models by oral administration and intrathecal injection [3], as well as having a strong analgesic effect when tested by the glacial acetic acid-, formalin-, and heat-induced pain models in mice [1].

Essential oils containing in borneol (1.8–18%, v/v) showed analgesic activity. For example, *Artemisia ludoviciana* essential oil, rich in borneol (18%, v/v), had a strong analgesic activity in formalin- and heat-induced mouse pain models [4]. *Rosmarinus officinalis* L. essential oil, of which the main components are camphene (11.5%, v/v), β-pinene (12%, v/v) and borneol (4.9%, v/v), had a good analgesic effect on arthritic rats [5]. Lavender essential oil, rich in linalool (32.5%, v/v), limonene (6.5%, v/v) and borneol (1.8%, v/v), had an analgesic effect in the formalin-induced mouse pain model, similar to the positive control tramadol [6]. Essential oils similar in components to BEO, including linalool, limonene, sabinene, β-caryophyllene, and caryophyllene oxide also showed analgesic activity. For example, *Zanthoxylum schinifolium* essential oil, of which the main components are linalool (32.5%, v/v), limonene (15.3%, v/v) and sabinene (9.2%, v/v), had analgesic activity in the glacial acetic acid- and heat-induced mouse pain models [7]. *Maqian* essential oil, of which the main components are limonene (67.1%, v/v) and linalool (3%, v/v), also had analgesic effects [8]. Essential oil from *Vitex agnus-castus*, containing limonene (10.3%, v/v), β-caryophyllene (6.9%, v/v) and sabinene (5.3%, v/v), showed analgesic activity in the formalin-induced mouse pain model [9]. *Hyptis pectinata* (L.) Poit essential oil, rich in β-caryophyllene (40.9%, v/v) and caryophyllene oxide (38.1%, v/v), had significant analgesic effects in the glacial acetic acid and heat-induced pain models in mice [10]. All these researches showed that analgesic activity not only from borneol, but also from other components.

Acute or chronic inflammation and pain are routinely treated with analgesic anti-inflammatory drugs, such as non-steroidal anti-inflammatory drugs (NSAIDs) and opioids [11], but these drugs have adverse side effects. NSAIDs have adverse effects on the gastrointestinal tract, liver, kidney, and central nervous system, whereas opioids can cause itching, constipation, nausea, addiction, and even fatal respiratory depression [12]. Those side effects have drawn attention to the benefits of topical medications. Topical analgesics, such as sprays, cream or gels, are applied to the skin over the painful area, the advantage being that the concentration of the drug in the plasma is greatly reduced, thereby reducing the incidence of systemic adverse reactions. In addition, topical analgesics also have a lower risk of drug-drug interactions, which is particularly important for the elderly and people taking multiple drugs [13]. Therefore, it is imperative to develop natural, safe, and convenient topical anti-inflammatory analgesic preparations.

In the present study, we hypothesized that BEO has an analgesic effect. To prove this hypothesis, safety of BEO for topical use was evaluated. The acute oral toxicity of BEO in mice and its capacity for skin and eye irritation were determined, and a mouse glacial acetic acid-induced pain model was used to evaluate the analgesic effect, as well as the expression of related pain mediators in serum. This information is expected to be helpful to the potential application of BEO in topical analgesic and other health products.

## 2. Materials and methods

### 2.1. Materials

BEO was provided by *Chunjingziran* Biotechnology Co., Ltd. (Zhejiang, China).

Glacial acetic acid (Sinopharm Group Co., Ltd, Shanghai, China), Caprylic/Capric triglyceride (GTCC) was from CRODA (Snaith, UK). Ketoprofen gel was from A. Menarini Manufacturing Logistics and Services (Florence, Italy). Prostaglandin E_2_ (PGE_2_) and transient receptor potential melastatin-8 (TRPM8) in mouse serum were determined by enzyme-linked immunosorbent assay (ELISA) kits (SenBeiJia Biotechnology Co., Ltd, Nanjing, China).

### 2.2. Chemical composition analysis of BEO

Gas chromatography/mass spectrometry (GC-MS) was carried out using an Agilent 7890B gas chromatograph (Agilent, U.S.A.) equipped with a DB-Wax fused silica capillary tubes column (30 m × 0.25 mm × 0.25 μm) that was directly connected to a Time of Flight Mass Spectrometer (Pegasus BT, USA). The heating program was set as follows: initial temperature 45°C (2 min), heating rate 8 /min, to 230°C (10 min), carrier gas (He) flow rate 1.0 mL/min, inlet temperature 250°C. The extraction head was desorbed at 250°C for 3 minutes before injection and the liquid sample (0.5 μL) was directly injected. MS conditions were set as follows. Electron impact (EI) ionization, interface temperature 250°C, ion source temperature 200°C, emission current 100 μA, electron energy 70 eV, detector voltage 1000 V, scanning mass range 33–450 amu [2].

GC-FID analysis was accomplished on a Shimadzu GC2010 equipped with a flame ionization detector (FID). The detector temperature was 230°C. The other analytic conditions including the column type and column temperature, the injector temperature, carrier gas and the linear velocity were the same as those of GC–MS analysis, the peak area from the chromatogram converted to mass using an internal standard (1-octanol) [14]. The components were divided into four categories (monoterpenes, oxygenated monoterpenes, sesquiterpenes, and oxygenated sesquiterpenes), and the correction factor for each category was calculated by the representative substance (borneol, camphor, α-pinene, β-caryophyllene). The mixtures of n-alkanes (C6-C26) were injected with the same program to calculate the retention index (RI) for each peak. RIs were documented in the National Institute of Standards and Technology (NIST) WebBook Database (https://webbook.nist.gov/chemistry/) [15].

### 2.3. Experimental animals

Experimental animal procedures were approved by the Ethics Committee of the Experimental Animal Center of Jiangnan University (Wuxi, Jiangsu Province, JN. No. 20181230i0780117 [288], JN. No. 20180615M0880820 [155]). Care and use of laboratory animals proceeded in accordance with national and international guidelines (Directive 2010/63/EU).

### 2.4. Safety evaluation of BEO

#### 2.4.1. Acute oral toxicity of BEO

According to the China National Food Safety Standard [16], Horn’s method was used to determine the acute oral median lethal dose (LD_50_) of BEO. Fifty ICR (Institute of Cancer Research) mice were selected and randomly divided into five groups: ten in each group, half male and half female. Aliquots of BEO (1, 2.15, 4.64, and 10 mL) were made up to 20 mL with corn oil and mixed thoroughly to prepare four concentrations of BEO for the biological assays. The experimental animals were fasted for 12 h before the experiment, but drinking water was not restricted. Test BEO mixtures were administrated once, by oral gavage (20 mL/kg). The test animals were fasted for 1 to 2 h after exposure, and then observed for 14 d. The number of deaths, time of death, poisoning manifestations and weight of each animal were recorded daily. LD_50_ values were determined from the calculation table of Horn’s method [17,18].

#### 2.4.2. Assay of BEO skin and eye irritation

##### 2.4.2.1. Multiple-dose rabbit skin irritation test

Skin irritation tests were performed as described previously [19] with some modifications, 16 New Zealand rabbits, aged 4 weeks, weighing 1.5 to 2.5 kg were randomly divided into four treatment groups (25, 50, 70 and 100% BEO), four in each group, half male and half female. Twenty-four hours before the experiment, the fur was carefully shaved, over an area of about 3 × 3 cm, on both sides of the spine on the back of each experimental animal, taking care not to damage the skin. Only healthy, undamaged skin areas were used for the experiments. Each day, the relevant test substance for each group (0.2 mL) was slowly applied to the skin over an area of 2.5 × 2.5 cm, on one side of the spine. GTCC was applied on the other side as a control. Then, the shaved areas were covered with four layers of gauze and held in place with nonirritating tape and a bandage. The test substance and GTCC were removed 4 h after application. After 1 h, the sites were macro-pathologically examined for skin irritation [20].

Individual evaluation of test-sites was scored according to the Draize Scoring System [19] (Table S1), approximately 1 h after the removal of BEO during a 14-d experimental period. The skin irritation score was the daily points total (the average score of four rabbits) during the observation period (14 d) [20,21]. The degree of irritation was classified according to the descriptive rating for the mean dermal irritation score illustrated by Shara et al. (2005) [22].

###### 2.4.2.1.1 Single-dose skin irritation test

Rabbits were treated with BEO at different concentrations, as described in Section 2.4.2.1, but with a single dose. Individual evaluation of the test sites was performed after 1, 24, 48 and 72 h.

##### 2.4.2.2 Multiple-dose rabbit eye irritation test

Eye irritation tests were performed as described previously [19,21] with some modifications. Three test concentrations, which had not previously caused skin irritation (12.5, 25, and 50% BEO) were selected for eye irritation experiments. Twelve four-week-old New Zealand rabbits were randomly divided into three groups of four, half male and half female. Each day, a test sample (0.1 mL), was dripped into the right eye of each rabbit, and the eyelid was gently held closed for 3 to 5 s, GTCC was added to the left eye as a control. The animals were observed at 1 h post-treatment for corneal opacity, iritis, or conjunctival irritation. Individual eye evaluation was scored according to the Scoring System (Table S2) in the China national standard [21]. Rabbits were repeatedly treated with BEO at different concentrations, once per day, for a consecutive 14 d. The eye irritation score was the daily points total (the average score of four rabbits) during the observation period (14 d) [21]. After the experiment, the eyeball was taken for histopathological examination.

###### 2.4.2.2.1 Single-dose eye-irritation test

Rabbits were treated with BEO at different concentrations, as described in Section 2.4.2.2, but with a single dose. Individual evaluation of the eyes was repeated after 1, 24, 48, and 72 h.

### 2.5. BEO analgesic activity

#### 2.5.1. Glacial acetic acid-induced writhing test

Mice (18–22 g) were randomly divided into seven groups: ten in each group, half male and half female. BEO (2.6, 4.4, and 6.1 g/kg) was topically applied daily to the abdomen, ketoprofen gel (10 g/kg) was applied to the positive control group and GTCC (8 g/kg) was applied to the negative control group. The treatments were applied daily for 6 d. Then, 20 min after the last treatment, each mouse (except in the control group) was intraperitoneally injected with 0.6% glacial acetic acid (0.2 mL/20 g). The number of writhing movements within 20 min was recorded for each mouse and pain inhibition was calculated [12]. Pain inhibition was calculated as follows:

$$\%\;Pain\;inhibition=100\;\;\left(1-\frac{A2}{A1}\right)$$

A1: number of writhing movements in the model group within 20 min, A2: number of writhing movements in the sample group within 20 min.

After the mouse was anesthetized, blood (0.7 mL) was collected from the venous sinus of the eyeball, and plasma was isolated by centrifuging the whole blood at 2500 g for 10 min. It was then stored at −80°C until needed. The levels of PGE_2_ and TRPM8 in the samples were evaluated by ELISA.

#### 2.5.2. Grip-strength measurement on mice

To verify whether BEO had any side effects on motor function or motor coordination, the grip-strength test was performed. A grip strength meter (YLS-13A, Yiyan Technology Development Co., Ltd., Shandong, China) was used to evaluate forelimb grip strength [2]. Mice were lifted by their tails, so their forepaws could grip the wire of the strength meter, and then gently pulled back with their tails parallel to the surface of the table until they lost their grip on the wire [2]. The maximum force was recorded in grams-force (gf). Three tests were performed on each mouse, and the average score was used for statistical analysis [23].

### 2.6. Histopathological analysis of tissue samples

Fresh rabbit skin- and eye-tissue samples were fixed with 4% paraformaldehyde for 24 h, dehydrated, embedded in paraffin, sectioned, and stained (hematoxylin and eosin, HE) [2]. Histopathology was observed with an inverted fluorescence microscope.

### 2.7. Data analysis

Prism 6 software (GraphPad, San Diego, CA) and OriginLab-9.0s (Origin Lab, Northampton, MA) were used for data analysis and plotting. The results are expressed as the mean ± standard deviation. The data were analyzed using one-way analysis of variance with Dunnett’s multiple comparisons test; *p < 0.05, **p < 0.01, ***p < 0.001, ****p < 0.0001 were considered as statistically significant.

## 3. Results and discussion

### 3.1. Chemical composition of BEO

Through GC-MS/GC-FID analysis, BEO contained 30 components (Table S3) accounting for 98.9% (v/v) of the total essential oil, the main components of BEO were borneol (175.5 mg/mL), α-pinene (116.7 mg/mL), l-Phellandrene (115.7 mg/mL), limonene (91.8 mg/mL), camphor (89.2 mg/mL), β-caryophyllene (38.1 mg/mL), β-pinene (36.7 mg/mL) and linalool (3.3 mg/mL) [2] (Table S3), these key compounds were similar to that obtained by neutral cellulase-assisted steam distillation in the previous study, such as borneol (11.7%), β-pinene (8.6%) and linalool (0.3%), although there were some differences in the number of compounds and their proportions [24], it may be caused by different extraction methods [25].

### 3.2. Acute oral toxicity

Borneol has potential pharmacological activity [1], but before use for pharmaceutical applications, BEO must be demonstrated to be safe for human use. Therefore, the acute oral toxicity of BEO was determined. All mice in the groups dosed at 10.0 mL/kg died within 24 h, but no death occurred in the 1 mL/kg dose group, during the 14-d observation period. Gross dissection of the dead animals did not reveal any obvious lesions in the tissues, or organs. The LD_50_ of BEO for female mice was 2749 mg/kg and that for male mice was 5081 mg/kg, which are classified as low toxicity and nontoxic, respectively, according to Horn’s LD_50_ appendix [16,17]. A gender difference in toxicity is commonly observed, available literature indicates that females are often more sensitive than males when acute toxicity differences do exist [26], this is also consistent with our research. For example, the acute oral LD_50_ of catnip oil [(E, Z)-nepetalactone (90%), caryophyllene (10%)] was 3160 mg/kg for female and 2710 mg/kg for male mice [19].

**Figure 1.**
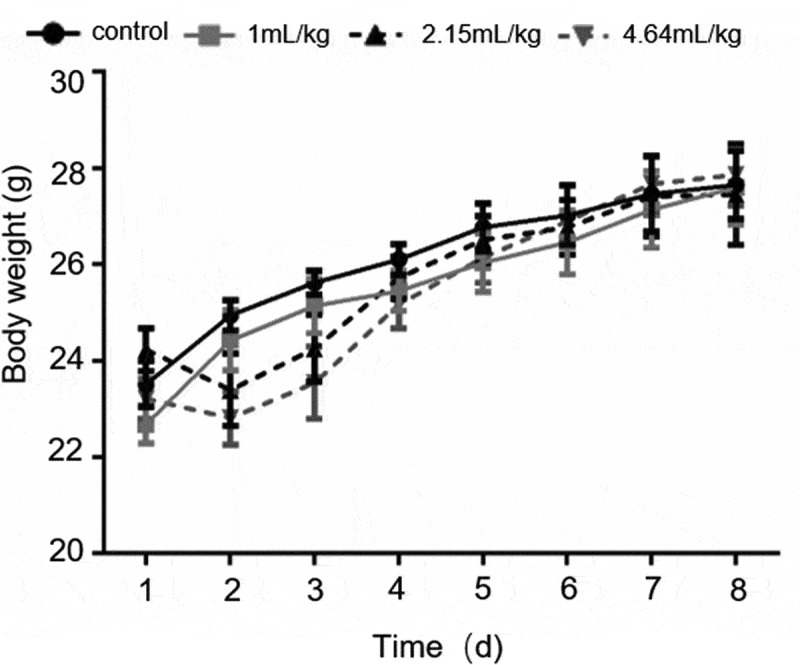
Bodyweight of mice treated orally with BEO

There was no significant difference in body weight between the surviving mice and the control group mice. The weight of the surviving mice in the medium-dose groups (2.15 and 4.64 mL/kg) decreased slightly during the first 3 d (Figure 1), and then returned to normal thereafter. This may have been caused by the mice being put off their food, at the early stage of intragastric administration.

(Data are expressed as mean ± standard deviation, n = 10)

Several studies showed that some monoterpenes (e.g., pulegone, menthofuran, camphor, and limonene) and sesquiterpenes (e.g., zederone, germacrone) exhibited liver toxicity [27]. BEO also contains camphor and limonene, their content may be related to the toxicity of BEO.

### 3.3. Skin irritation testing of BEO

#### 3.3.1. Acute skin irritation

Similar to other essential oils, BEO is expected to be used mainly as an external treatment. Therefore, it is important to assess whether BEO causes acute, or chronic skin irritation.

According to the Draize Scoring System (Table S1), the single skin irritation response score and skin stimulus intensity score of the 25, 50, and 70% BEO groups, were all 0 points, which means they were nonirritating, whereas the skin irritation intensity score of 100% BEO was 0.25. However, only one of the four rabbits had erythema on the skin, which recovered within 24 h and was nonirritating (Table S1). Therefore, the above concentrations of BEO will not cause acute skin irritation to the rabbit. As such, it is safe for topical use.

#### 3.3.2. Chronic skin irritation

**Figure 2.**
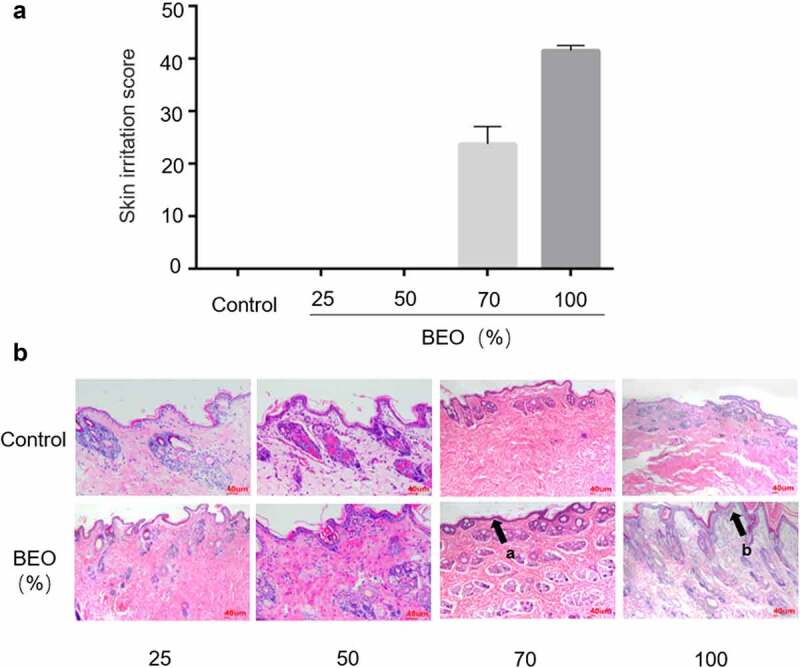
Effect of topical treatment with BEO (25, 50, 70, and 100%) on multiple-dose skin irritation. A: The skin irritation score of mice after topical application of BEO (25, 50, 70, and 100%). B: Skin sections in the group were treated with (25, 50, 70, and 100%) on day 14. (a) Epidermal thickening, (b) Epidermal thickening with keratinocytes proliferation

If BEO is to be developed as a topical skin treatment, for continuous use, its capacity for chronic skin irritation needs to be evaluated. After 14 consecutive days of skin application, the New Zealand rabbit’s skin part performed normal (Figure 2) and none died. The skin of the 25% and 50% BEO application groups was normal and there was no significant difference between the administration and control sites. Histopathological examination showed that the skin tissue was intact. There was no structural disorder, edema, or thickening of the skin’s spinous layer (Figure 2). In the 70% BEO group, the skin was slightly swollen and chapped after 5 d administration and desquamated after 10 d, but the chapping and desquamation improved after 14 d. Histopathological examination of the skin revealed slight epidermal thickening (black arrow (a) in Figure 2(b)) and the boundary between the epidermal layer and the superficial dermis was clearly defined (Figure 2(b)). In the 100% BEO group, after 5 d administration, the skin at the administration site appeared red, swollen, and cracked; desquamation of the skin was visible after 10 d and after 14 d, the swelling, chapping, and desquamation at the test site worsened. Histopathological examination showed significant epidermal thickening with keratinocytes (black arrow (b) in Figure 2(b)) and the boundary between the epidermal layer and the superficial dermis was clearly defined. Irritation scores were calculated according to the Draize Scoring System and Chinese national standard [21], which stipulates that if the score exceeds 30, the test substance is considered to cause skin irritation. The scores of the 25, 50, and 70% BEO groups were 0, 0, and 24 respectively, which indicated that there was no significant irritation (Figure 2(a)). The score of the 100% BEO group was 42, indicating that pure BEO caused skin irritation. Therefore, BEO is safe for topical use at less than 70% concentration. Similarly, continuous application of *Lippia sidoides* Cham. essential oil (main component thymol, 71%) for 7 d caused skin irritation in mice at concentrations of 12, 25, 50, and 100% [28].

### 3.4. Eye irritation by BEO

#### 3.4.1. Acute eye irritation testing

According to the requirements of the Chinese National Standard [21], substances at concentrations determined to cause skin irritation should not be used for eye irritation testing. Therefore, according to the skin test results above, single-dose eye testing was limited to 12.5, 25 and 50% concentrations. At all three concentrations, the eye irritation intensity scores were 0 (Table S2), indicating that BEO is safe below 50% concentration.

#### 3.4.2. Continuous eye irritation testing

**Figure 3.**
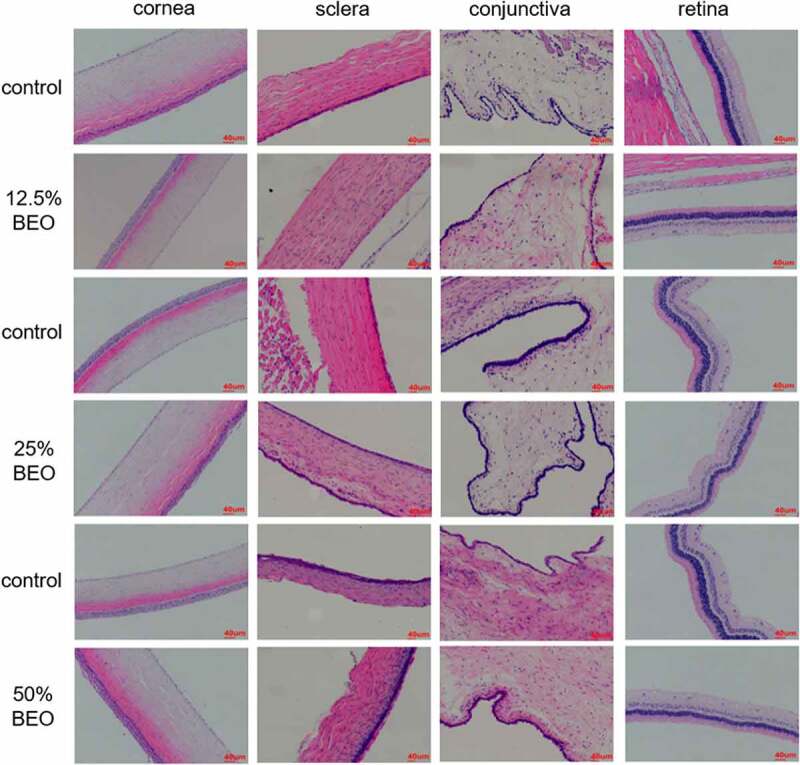
Effect of topical treatment with BEO (12.5, 25, and 50%) on multiple-dose eye-irritation

For continuous eye irritation experiments, 12.5, 25, and 50% BEO were administered daily for 14 d and the eye irritation intensity scores of all three groups were 0 (Table S2). Compared with the control group, the results from all three groups were similar. The corneas were not turbid, the conjunctivas were free of congestion, edema and secretions, the pupils on both sides were round and the same size and the light reflection was good, indicating the absence of eye irritation. Further histopathological examination of the treatment groups (Figure 3) showed that the corneal structure of the eye was complete. The boundaries were clear and there was no damage, epithelial hyperplasia, white spots, or inflammation. The scleral structure was complete, with clear boundaries, no visible thickening, no congestion, and no lymphocyte infiltration. The conjunctival tissue structure was complete, without epithelial hyperplasia, vasodilatation, congestion, or inflammatory cell infiltration. Each layer of the retina had a complete structure and clear boundaries, and there was no bleeding, congestion, scarring, or inflammatory cell infiltration.

The absence of eye-irritation by BEO was similar to catnip oil [19], which also showed no eye irritation. In this study, the macroscopic and histopathological examinations were combined to verify the safety of BEO to the eye. This provided a secure basis for the future development of safe facial products.

### 3.5. Analgesic effect of BEO

#### 3.5.1. Glacial acetic acid-induced writhing test

**Figure 4.**
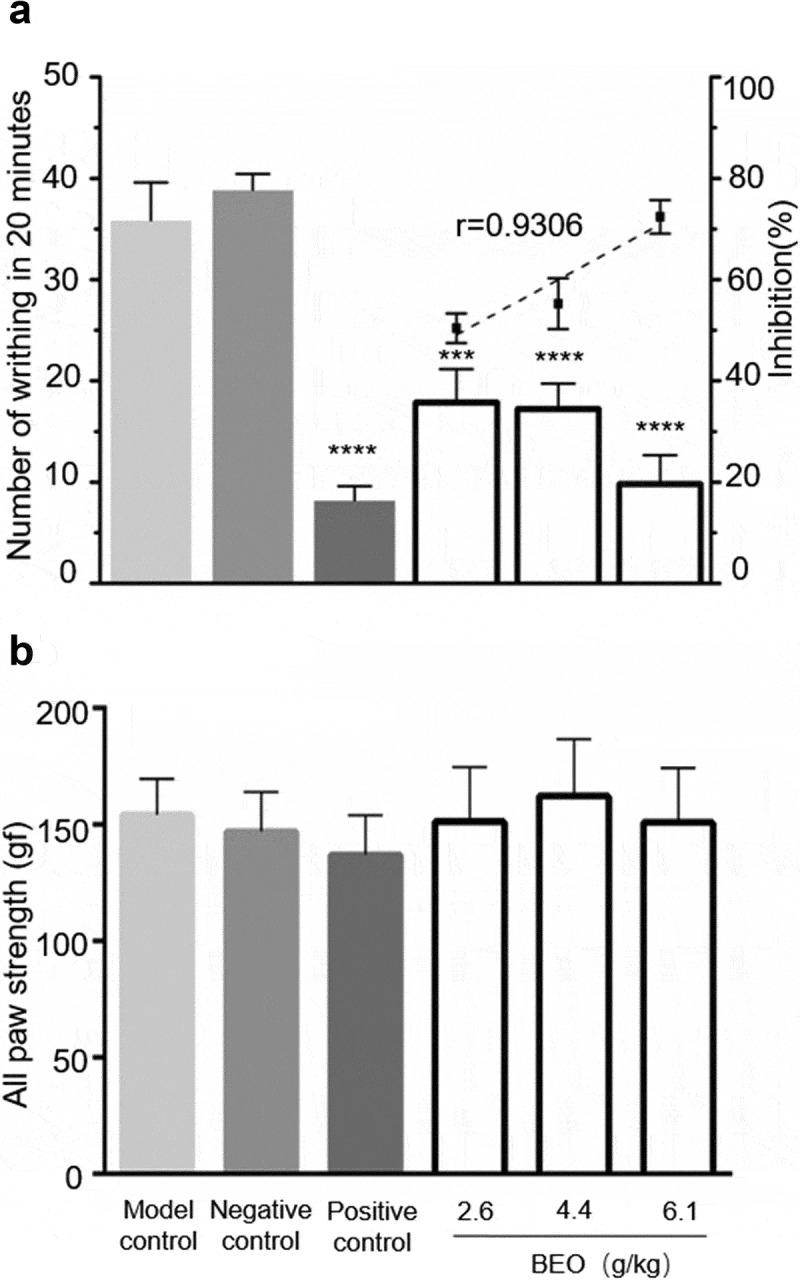
Effect of BEO (2.6, 4.4, and 6.1 mg/kg) on a number of writhing movements induced by glacial acetic acid. (a) number of writhing movements in 20 min, (b) Paw grip-strength (gf). Data are expressed as mean ± standard deviation (n = 10), ***p < 0.001, ****p < 0.0001, compared with the model control

When BEO was topically applied to the abdomen of mice for six consecutive days, the number of writhing movements caused by glacial acetic acid was significantly reduced. Compared with that of the model group, the number of writhing movements of all the BEO treatment groups and the positive control group were markedly reduced over 20 min (*p* < 0.001), in a dose-dependent manner (r = −0.9006) (Figure 4(a)). The glacial acetic acid-induced mouse writhing model is based on intraperitoneal injection of glacial acetic acid, which generates a periodic and characteristic stretching (peristaltic) movement, manifesting as limb extension and abdominal recession. It is widely used to screen new analgesics, including for neuropathic and inflammatory pain, and has often been used to evaluate the analgesic effects of NSAIDs and Cyclooxygenase-2 inhibitors [1].

Studies have shown that natural borneol has a significant analgesic effect in glacial acetic acid- and heat-induced pain models in mice [1]. α-pinene and linalool are proven to have significant anti-inflammatory and analgesic effects in xylene- and formalin-induced pain models in mice [29]. l-Phellandrene has a significant analgesic effect in formalin-, carrageenan-, glacial acetic acid- and heat-induced pain models in mice [30]. β-caryophyllene also has a significant analgesic effect in formalin-, and heat-induced pain models in mice [31]. Some borneol-rich essential oils have analgesic effects, such as those from *Blumea balsamifera* (L.) DC. (borneol 33.22%) [32], *Artemisia ludoviciana* (borneol 18.00%) [4], *Rosmarinus officinalis* L. (borneol 4.85%) [5] and lavender (borneol 1.76%) [6]. Since borneol, β-caryophyllene α-pinene was the major compound in BEO, it was considered worthwhile to evaluate the analgesic effect of BEO.

Previous studies reported that intrathecal or intraperitoneal injection of borneol had significant analgesic effects in several different pain models [1,3]. There was no significant difference (*p* > 0.05) in grip strength between mice with BEO topically applied for 6 d and the control group (Figure 4(b)), indicating that the application of BEO did not affect motor function. This is consistent with a report on the intraperitoneal injection of borneol [1], showing the absence of any side effects.

#### 3.5.2. Expression of pain and related mediators in mouse serum

**Figure 5.**
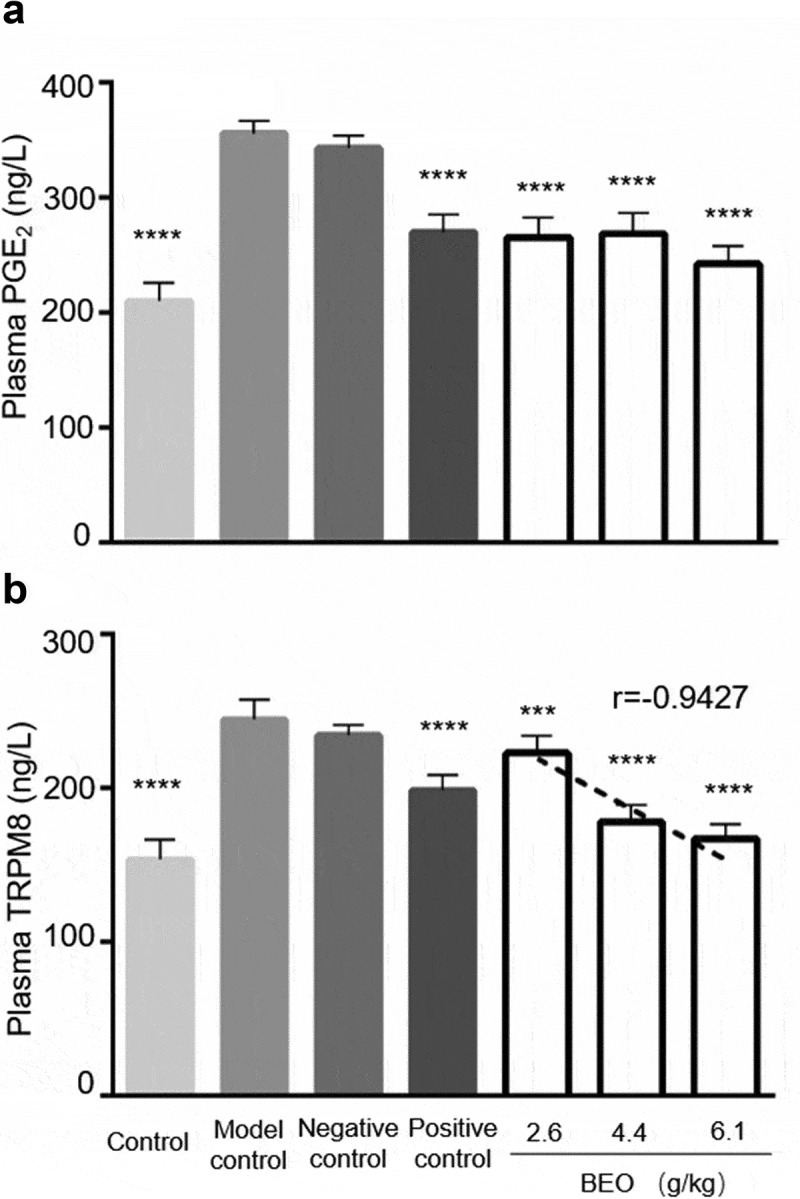
Effect of BEO on serum expression of PGE_2_ (a) and TRPM8 (b) in the mouse glacial acetic acid-induced writhing pain model. Data are expressed as mean ± standard deviation (n = 10), ***p < 0.001, ****p < 0.0001, compared with model control

To study the analgesic effect of BEO at the molecular level, the pain factors PGE_2_ and TRPM8 in the serum of mice from the glacial acetic acid-induced writhing model were assayed. Serum expression of PGE_2_ and TRPM8 in the BEO treatment group and positive control groups was significantly reduced (Figure 5), and the reduced expression of TRPM8 was dose-dependent (r = −0.9427). PGE_2_ is an important mediator of pain and inflammation; high levels of PGE_2_ expression have been observed in many disease states and NSAIDs reduce pain by inhibiting PGE_2_ production [33]. In neuropathic pain, pain neurons are stimulated by increased TRPM8 expression in nociceptive neurons, which leads to neuron excitation, cold tactile hypersensitivity, and finally pain [34]. During chronic pain, TRPM8 expression is up-regulated, which leads to hyperalgesia and hypersensitivity in pain-sensing sites [35].

As the BEO concentration increased, the expression of TRPM8 gradually decreased, suggesting that the analgesic effect of BEO is related to the down-regulation of PGE_2_ and TRPM8 expression, which is consistent with previous reports. For example, the analgesic and anti-inflammatory effects of *Lippia gracilis* essential oil (rich in carvacrol and cymene) [36] and *Hyptis pectinata* (L.) Poit essential oil (rich in β-caryophyllene and caryophyllene oxide) [10] was mediated by inhibition of NO and PGE_2_ production. The analgesic effect of topically applied borneol was mediated by TRPM8 [11], and its selective antagonists could reduce both acute and chronic pain [37]. This study confirmed that the analgesic effect of BEO was achieved by down-regulating PGE_2_ and TRPM8 in mice.

## 4. Conclusion

BEO was found to have a significant analgesic effect and a clear dose-response relationship when tested in a glacial acetic acid-induced mouse pain model, and its analgesic effect was mediated by down-regulating PGE_2_ and TRPM8 in this species (mouse). The analgesic effect observed for BEO is not only consistent with that of borneol, but also with that of other essential oils with a similar composition to BEO. There was a gender difference in mice for the acute oral LD_50_, i.e., low-toxicity to female mice and practically nontoxic to male mice, and there was no acute skin or eye irritation when pure BEO was applied directly at concentrations less than 50%. Moreover, these findings indicate that BEO is safe to use in both internal and external treatments, and appears to have great potential for application in topical analgesics and other health products.

## Supplementary Material

Supplemental Material

## ABBREVIATIONS

*Cinnamomum camphora* chvar. Borneol (*C. camphora*), Borneol essential oil (BEO), non-steroidal anti-inflammatory drugs (NSAIDs), gas chromatography-mass spectrometry (GC-MS), triglyceride caprylate (GTCC), grams-force (gf), median lethal dose (LD_50_), Chinese National Standard (GB), prostaglandin E_2_ (PGE_2_), transient receptor potential melastatin-8 (TRPM8), Enzyme-linked immunosorbent assay (ELISA).
