# Supplementary material for: Evaluation of the analgesic potential and safety of Cinnamomum camphora chvar. Borneol essential oil
Source: Bioengineered. 2021 Dec 9;12(2):9860–71. doi: 10.1080/21655979.2021.1996149 (PMC8810075; doi:10.1080/21655979.2021.1996149)
Supplement: Supplemental Material [file KBIE_A_1996149_SM9171.zip › Table S1clean.docx]

Table S1. Scoring criteria and dermal reactions in the skin irritation study of BEO.

| Scoring criteria | |  | Average score | | | | | | | | | | | | |
| --- | --- | --- | --- | --- | --- | --- | --- | --- | --- | --- | --- | --- | --- | --- | --- |
| Items | Score |  | Single dose ^a^ | | | | |  | | Multi-dose ^b^ | | | | |  |
|  |  |  | BEO (%) | | | |  | | BEO (%) | | | | |  |  |
|  |  |  | 30 | 50 | 70 | 100 |  | | 30 | | 50 | 70 | 100 |  |  |
| **I Erythema formation** | |  |  |  |  |  |  | |  | |  |  |  |  |  |
| No erythema | 0 |  | 0 | 0 | 0 | × |  | | 0 | | 0 | × | × |  |  |
| Very slight erythema (barely perceptible with no defined edges) | 1 |  | / | / | / | 0.25 |  | | / | | / | 1 | × |  |  |
| Slight erythema (pale red in colour with definable edges) | 2 |  | / | / | / | / |  | | / | | / | / | 2 |  |  |
| Moderate to severe erythema (defined by colour with well-defined area) | 3 |  | / | / | / | / |  | | / | | / | / | / |  |  |
| Severe erythema (coloured crimson red) | 4 |  | / | / | / | / |  | | / | | / | / | / |  |  |
| The sum of the average score ^c^ | |  | - | - | - | - |  | | 0 | | 0 | 12 | 22 |  |  |
| **II Edema formation** | |  |  |  |  |  |  | |  | |  |  |  |  |  |
| No oedema | 0 |  | 0 | 0 | 0 | 0 |  | | 0 | | 0 | × | × |  |  |
| Very slight oedema (barely perceptible with no defined edges) | 1 |  | / | / | / | / |  | | / | | / | 1 | × |  |  |
| Slight oedema (edges of area well defined by definite raising) | 2 |  | / | / | / | / |  | | / | | / | / | 2 |  |  |
| Moderate oedema (raised approximately 1 mm) | 3 |  | / | / | / | / |  | | / | | / | / | / |  |  |
| Severe oedema (raised > 1 mm and extending beyond area of exposure) | 4 |  | / | / | / | / |  | | / | | / | / | / |  |  |
| The sum of the average score ^c^ | |  | - | - | - | - |  | | 0 | | 0 | 12 | 20 |  |  |

Note: ^a^: represented the average score of 4 rabbits. ^b^: represented the average score during 14 d for each rabbit. ^c^: represented the sum of the average score of each rabbit with multi-dose for 14 d. “/”：represented no reaction. “×”: represented reaction. “-”: represented no total score. The skin irritation index is the sum of the average of the two items.
