# Supplementary material for: Evaluation of the analgesic potential and safety of Cinnamomum camphora chvar. Borneol essential oil
Source: Bioengineered. 2021 Dec 9;12(2):9860–71. doi: 10.1080/21655979.2021.1996149 (PMC8810075; doi:10.1080/21655979.2021.1996149)
Supplement: Supplemental Material [file KBIE_A_1996149_SM9171.zip › Table S2.docx]

Table S2. Scoring criteria and ocular reactions in the eye irritation study of BEO.

| Scoring criteria | |  | Average score | | | | | | |
| --- | --- | --- | --- | --- | --- | --- | --- | --- | --- |
| Items | Score |  | Single dose ^a^ | | |  | Multi-dose ^b^ | | |
|  |  |  | BEO (%) | | |  | BEO (%) | | |
|  |  |  | 12.5 | 25 | 50 |  | 12.5 | 25 | 50 |
| **Ⅰ *Cornea*** (A) Area of cornea involved | |  |  |  |  |  |  |  |  |
| No ulceration or opacity | 0 |  | 0 | 0 | 0 |  | 0 | 0 | 0 |
| > 0%, ≤ 25% | 1 |  | / | / | / |  | / | / | / |
| > 25%, ≤ 50% | 2 |  | / | / | / |  | / | / | / |
| > 50%, ≤ 75% | 3 |  | / | / | / |  | / | / | / |
| > 75%, ≤ 100% | 4 |  | / | / | / |  | / | / | / |
| Score = A× 3 Maximum total = 12 | |  | 0 | 0 | 0 |  | 0 | 0 | 0 |
| The sum of the average score ^c^ | |  | - | - | - |  | 0 | 0 | 0 |
| **ⅠⅠ *Iris***  (A) Scores | |  |  |  |  |  |  |  |  |
| Normal | 0 |  | 0 | 0 | 0 |  | 0 | 0 | 0 |
| Markedly deepened rugae, congestion, swelling, circumcorneal injection (any or all of these or combination thereof), iris still reacting to light (sluggish reaction is positive) | 1 |  | / | / | / |  | / | / | / |
| No reaction to light, haemorrhage, gross destruction (any or all of these) | 2 |  | / | / | / |  | / | / | / |
| Score = A× 3 Maximum total = 6 | |  | 0 | 0 | 0 |  | 0 | 0 | 0 |
| The sum of the average score ^c^ | |  | - | - | - |  | 0 | 0 | 0 |
| **III** ***conjunctiva*** (A) Redness (refers to palpebral and bulbar conjunctivae excluding cornea and iris) |  |  |  |  |  |  |  |  |  |
| Blood vessels normal | 0 |  | 0 | 0 | 0 |  | 0 | 0 | 0 |
| Some blood vessels definitely hyperaemic (injected above) normal | 1 |  | / | / | / |  | / | / | / |
| Diffuse, deeper crimson colour, individual vessels not easily discernible | 2 |  | / | / | / |  | / | / | / |
| Diffuse beefy red | 3 |  | / | / | / |  | / | / | / |
| Score = A× 3 Maximum total = 9 | |  | 0 | 0 | 0 |  | 0 | 0 | 0 |
| The sum of the average score ^c^ | |  | - | - | - |  | 0 | 0 | 0 |

Note: ^a^: represented the average score of 4 rabbits. ^b^: represented the average score during 14 d for each rabbit. ^c^: represented the sum of the average score of each rabbit with multi-dose for 14 d. “/”: represented no reaction. “-”: represented no total score.
