# Supplementary material for: Evaluation of the analgesic potential and safety of Cinnamomum camphora chvar. Borneol essential oil
Source: Bioengineered. 2021 Dec 9;12(2):9860–71. doi: 10.1080/21655979.2021.1996149 (PMC8810075; doi:10.1080/21655979.2021.1996149)
Supplement: Supplemental Material [file KBIE_A_1996149_SM9171.zip › Table S3.docx]

Table S3. Chemical composition of BEO.

| Classification | Compounds | Retention time（min） | Retention Indices | | Concentration (mg/mL) |
| --- | --- | --- | --- | --- | --- |
|  |  |  | Measured | Documented ^1^ |  |
| Monoterpenes | α-Pinene | 3.61 | 931 | 934 | 116.7 |
|  | Camphene | 4.21 | 948 | 948 | 46.7 |
|  | β-Pinene | 4.86 | 981 | 981 | 36.7 |
|  | Sabenene | 5.10 | 970 | 972 | 9.2 |
|  | 3-Carene | 5.56 | 1005 | 1009 | 3.5 |
|  | l-Phellandrene | 5.87 | 1003 | 1003 | 115.7 |
|  | α-Terpinene | 6.09 | 1014 | 1014 | 3.5 |
|  | Limonene | 6.44 | 1027 | 1030 | 91.8 |
|  | cis-Ocimene | 7.07 | 1027 | 1027 | 0.8 |
|  | γ-Terpinene | 7.23 | 1050 | 1050 | 5.9 |
|  | Ocimene | 7.36 | 1042 | 1042 | 3.4 |
|  | Cymene | 7.72 | 1032 | 1032 | 36.2 |
|  | Terpinolene | 7.91 | 1078 | 1078 | 11.3 |
| Oxygenated monoterpenes | 1,8-Cineole | 6.56 | 1011 | 1015 | 30.2 |
|  | Linalool | 13.24 | 1084 | 1084 | 3.3 |
|  | Terpinen-4-ol | 14.42 | 1424 | 1421 | 7.9 |
|  | Borneol | 16.60 | 1698 | 1698 | 175.5 |
|  | Neryl alcohol | 18.65 | 1785 | 1785 | 1.1 |
| Sesquiterpenes | β-Elemene | 14.09 | 1387 | 1387 | 2.2 |
|  | β-Caryophyllene | 14.24 | 1389 | 1389 | 38.1 |
|  | Humulene | 15.75 | 1179 | 1179 | 16.8 |
|  | Germacrene | 19.04 | 1802 | 1802 | 2.2 |
|  | α-Selinene | 16.88 | 1664 | 1664 | 1.1 |
| Oxygenated sesquiterpenes | Camphor | 12.64 | 1148 | 1146 | 89.2 |
|  | Caryophyllene oxide | 21.97 | 1565 | 1560 | 3.8 |
|  | Spathulenol | 23.86 | 1617 | 1608 | 4.7 |
|  | Juniper camphor | 25.22 | 1691 | 1691 | 1.1 |
|  | Humulene epoxide II | 22.80 | 1391 | 1400 | 1.4 |
| Others | Bicyclogermacrene | 17.09 | 1495 | 1493 | 10.1 |
|  | Methyl eugenol | 22.56 | 1589 | 1589 | 1.2 |

BEO: *Cinnamomum camphora* chvar. *Borneol* essential oil; ^1^: Retention indices documented in the National Institute of Standards and Technology (NIST) WebBook Database (https://webbook.nist.gov/chemistry/)
